# Hydrodeoxygenation of Levulinic Acid to γ-Valerolactone over Nano-β Zeolite-Supported Co–Ni Catalysts

**DOI:** 10.3390/molecules31214015

**Published:** 2026-11-06

**Authors:** Margarita Popova, Manuela Oykova, Momtchil Dimitrov, Yavor Mitrev, Pavletta Shestakova, Ágnes Szegedi

**Affiliations:** 1 Institute of Organic Chemistry with Centre of Phytochemistry, Bulgarian Academy of Sciences, Acad. G. Bonchev Str., Bl. 9, 1113 Sofia, Bulgaria; manuela.oykova@orgchm.bas.bg (M.O.); momtchil.dimitrov@orgchm.bas.bg (M.D.); yavor.mitrev@orgchm.bas.bg (Y.M.); pavletta.shestakova@orgchm.bas.bg (P.S.); 2 National Centre of Excellence Mechatronics and Clean Technologies, 1756 Sofia, Bulgaria; 3 HUN-REN Research Centre for Natural Sciences, Magyar tudósok körútja 2, 1117 Budapest, Hungary; szegedi.agnes@ttk.hu

**Keywords:** lignocellulosic biomass, levulinic acid, β-zeolite, γ-valerolactone, Co/Ni nanoparticles, solid-state NMR

## Abstract

Monometallic Co- and Ni- and bimetallic Co–Ni-containing catalysts supported on nanosized β-zeolite were prepared by a post-synthesis impregnation method and evaluated for the catalytic upgrading of biomass-derived levulinic acid (LA) to γ-valerolactone (GVL), an important platform molecule for the production of renewable fuels and value-added chemicals. The catalysts were characterized by XRD, N_2_ physisorption, H_2_-TPR, DR UV–Vis, solid-state NMR, XPS, FTIR of adsorbed pyridine and H_2_ chemisorption. Increasing metal loading resulted in the formation of larger NiO and Co_3_O_4_ domains, while metal incorporation modified the zeolite framework and reduced its Brønsted acidity. Among the studied catalysts, the bimetallic 15 wt.%Co–15 wt.%Ni/β-zeolite exhibited the highest catalytic activity, achieving complete LA conversion and 92% GVL yield at 150 °C and retaining 98% conversion after three reaction cycles. The enhanced catalytic behavior of bimetallic catalysts is attributed to the synergistic interaction between cobalt and nickel species, leading to the formation of highly dispersed metallic Ni/Co and probably Ni-Co alloy nanoparticles with improved reducibility and hydrogenation activity. In addition, the combination of metallic hydrogenation sites and acidic centers provided by the β-zeolite support promotes the consecutive hydrogenation required for efficient GVL formation. The results demonstrate that Co–Ni-modified β-zeolite is a promising low-cost alternative to noble-metal catalysts for the valorization of biomass-derived levulinic acid and highlight the importance of metal–metal and metal–support interactions in the design of efficient bifunctional catalysts for sustainable biomass conversion.

## 1. Introduction

Lignocellulosic biomass is considered one of the most promising renewable alternatives to fossil resources owing to its abundance, renewability, low cost, and widespread availability. The increasing global energy demand, depletion of fossil fuel reserves, and growing environmental concerns associated with greenhouse gas emissions have stimulated intensive research into the development of sustainable technologies for biomass conversion into fuels and chemicals [1,2,3]. Lignocellulosic biomass consists mainly of cellulose, hemicellulose, and lignin, which represent approximately 35–50%, 20–35%, and 10–25% of the biomass dry weight, respectively [4]. The efficient valorization of these components is a key challenge in modern biorefinery concepts aimed at replacing petroleum-derived products with renewable alternatives.

The hydrolysis of cellulose and hemicellulose into C6 and C5 monosaccharides constitutes a crucial step in biomass processing. These sugars can subsequently undergo dehydration, hydrogenation, oxidation, and other catalytic transformations to produce a variety of platform molecules, including furfural, 5-hydroxymethylfurfural (HMF), levulinic acid (LA), lactic acid, and sorbitol [5,6,7]. Among these compounds, levulinic acid has attracted particular attention because of its versatile chemical structure containing both carbonyl and carboxyl functional groups, which enable a broad range of chemical transformations. The U.S. Department of Energy has identified levulinic acid as one of the most important biomass-derived platform chemicals owing to its potential for the production of fuels, solvents, plasticizers, pharmaceuticals, polymers, and fuel additives [8,9].

Levulinic acid is commonly produced through acid-catalyzed hydrolysis and dehydration of cellulose-derived carbohydrates via the intermediate formation of 5-hydroxymethylfurfural. The commercial interest in levulinic acid has increased considerably in recent years due to the development of efficient production technologies and the growing demand for renewable carbon-based chemicals [10,11]. Among the numerous derivatives obtainable from levulinic acid, γ-valerolactone (GVL) has emerged as one of the most valuable products because of its favorable physicochemical properties and versatile applications. GVL is characterized by low toxicity, high thermal stability, high boiling point, and low vapor pressure, making it an attractive green solvent and a potential fuel additive [12,13,14]. Moreover, GVL can serve as an intermediate for the synthesis of liquid hydrocarbons, valerate esters, pentanoic acid derivatives, and polymer precursors, thus playing a significant role in integrated biorefinery schemes [15,16].

The catalytic hydrogenation of levulinic acid to γ-valerolactone is recognized as one of the most efficient routes for the valorization of biomass-derived feedstocks. The reaction generally proceeds through the hydrogenation of the carbonyl group followed by intramolecular esterification and dehydration steps, resulting in the formation of the five-membered lactone ring [17]. Both homogeneous and heterogeneous catalytic systems have been investigated for this transformation. Although homogeneous catalysts often exhibit excellent catalytic activity and selectivity, their practical application is hindered by difficult catalyst recovery, limited recyclability, and environmental concerns associated with catalyst disposal [18]. Consequently, heterogeneous catalysts have attracted considerable attention because they facilitate catalyst separation, recycling, and implementation in continuous-flow processes.

Numerous heterogeneous catalytic systems have been developed for LA hydrogenation. Supported noble metals such as Ru, Pd, Pt, and Ir have demonstrated excellent catalytic performance due to their superior hydrogen activation capability and high intrinsic activity [19,20,21]. In particular, ruthenium-based catalysts supported on carbon, alumina, silica, and zeolites frequently achieve nearly complete levulinic acid conversion and γ-valerolactone selectivity exceeding 95% under relatively mild reaction conditions [22,23]. However, the scarcity and high cost of noble metals, together with possible metal leaching and catalyst deactivation during long-term operation, limit their industrial applicability.

To overcome these drawbacks, increasing attention has been devoted to catalysts based on earth-abundant transition metals such as Ni, Cu, Co, and Fe [24,25,26,27,28,29,30]. Nickel-containing catalysts are particularly attractive due to their strong hydrogenation activity and relatively low cost. Copper-based catalysts exhibit high selectivity toward carbonyl hydrogenation while suppressing undesired side reactions, whereas cobalt-containing materials have shown promising activity in both hydrogenation and transfer hydrogenation processes [28]. Recent studies have demonstrated that synergistic interactions between Ni and Cu can improve metal dispersion, reducibility, and catalytic stability, resulting in enhanced performance in levulinic acid hydrogenation [31].

The catalytic behavior of heterogeneous systems is strongly influenced by the nature of the support. Besides providing high surface area for metal dispersion, the support can affect metal reducibility, particle size, hydrogen adsorption properties, and acid-base characteristics [22]. In the hydrogenation of levulinic acid, acidic sites are known to facilitate the cyclization and dehydration steps required for γ-valerolactone formation. Therefore, bifunctional catalysts possessing both metallic and acidic active sites are considered particularly effective [23].

Among various supports, zeolites have attracted significant interest because of their high surface area, well-defined pore structure, tunable acidity, and excellent thermal and hydrothermal stability [30]. β-Zeolite, in particular, possesses a three-dimensional pore system and a suitable distribution of Brønsted and Lewis acid sites, which can promote the formation of γ-valerolactone while simultaneously stabilizing highly dispersed metal particles. The incorporation of transition metals into zeolite structures by ion exchange, impregnation, or deposition methods enables the preparation of bifunctional catalysts combining hydrogenation and acid functionalities within a single material. Such catalysts have shown promising performance in the conversion of biomass-derived oxygenates and are considered attractive alternatives to noble-metal-containing systems.

Despite significant progress in the development of catalysts for levulinic acid hydrogenation, further improvements are required to increase catalytic activity, selectivity, and long-term stability while minimizing catalyst cost. In addition, optimization of reaction parameters such as temperature, hydrogen pressure, catalyst loading, reaction time, and space velocity remains essential for the development of economically viable processes. Therefore, the design of low-cost bifunctional catalysts based on transition metals and acidic zeolite supports continues to be an important research direction in sustainable biomass valorization.

In the present study, Ni- and Co-functionalized β-zeolites were prepared by post-synthesis impregnation and evaluated as catalysts for the hydrogenation of levulinic acid to γ-valerolactone. Particular attention was devoted to elucidating the influence of metallic and acidic active sites on catalytic performance, as well as to understanding the synergistic effects between nickel and cobalt species in the hydrogenation process.

## 2. Results and Discussion

**Figure 1:**
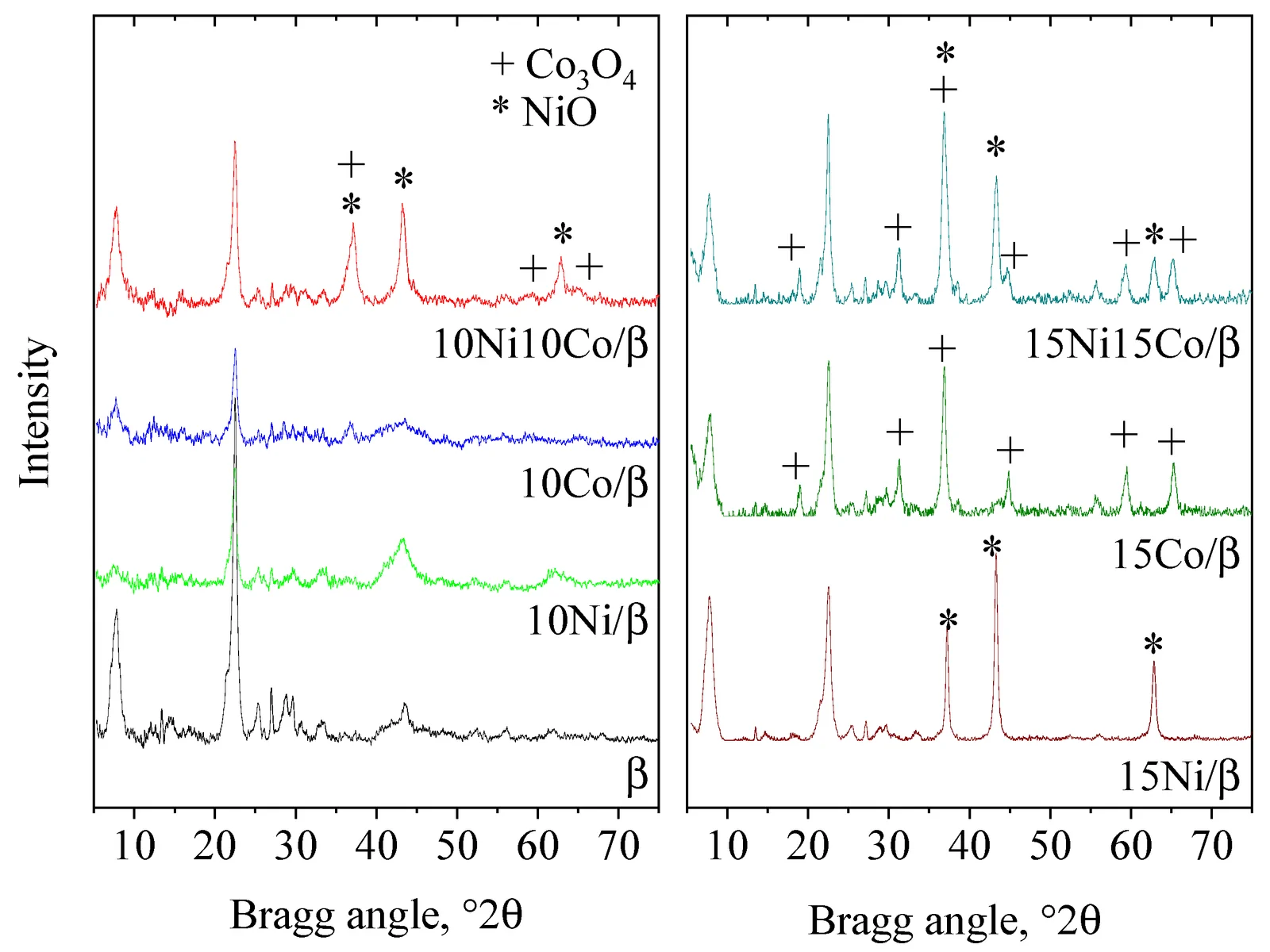
X-ray powder diffraction patterns of the parent and mono- and bimetallic Beta zeolites.

The crystalline phases of the as-prepared catalysts were investigated by X-ray powder diffraction (Figure 1). For the monometallic 10Co/β and 10Ni/β samples, only the reflections of zeolite Beta were observed. No traces of NiO and only very weak and broad reflections corresponding to the Co_3_O_4_ phase were detected. These results suggest that the metal oxides are highly dispersed on the external surface of the nanosized zeolite particles, while at least a fraction of the metal species strongly interacts with zeolite and is incorporated into the cationic position of the lattice. In contrast, at higher metal loadings, intense diffraction reflections corresponding to the respective metal oxides were observed, indicating the formation of separate oxide phases with relatively large crystallite sizes of approximately 35 nm and 26 nm for 15Ni/β and 15Co/β, respectively. Separate oxide phases were also detected in the bimetallic catalysts. On the 10Ni10Co/β catalyst, the lower intensity of the Co_3_O_4_ reflections compared with those of NiO suggests a stronger interaction of cobalt with the zeolite. It must be noted that in bimetallic catalysts the formation of NiCo_2_O_4_ spinel phase is highly probable; however, it cannot be distinguished from Co_3_O_4_ by the XRD method due to the very similar cation and unit cell size of Ni and Co. In contrast, both oxide phases exhibited more intense reflections in the 15Ni15Co/β catalyst. The crystallite sizes of the metal oxide nanoparticles, calculated using the Scherrer equation, are summarized in Table 1. In the 10Ni10Co/β catalyst, both oxides were highly dispersed, with crystallite sizes of approximately 10 nm. In comparison, larger crystallites were observed for the 15Ni15Co/β catalyst, with average sizes of 21 nm for Co_3_O_4_ and 14 nm for NiO.

These results indicate that increasing the metal loading promotes particle growth and agglomeration, particularly in the case of cobalt oxide.

**Figure 2:**
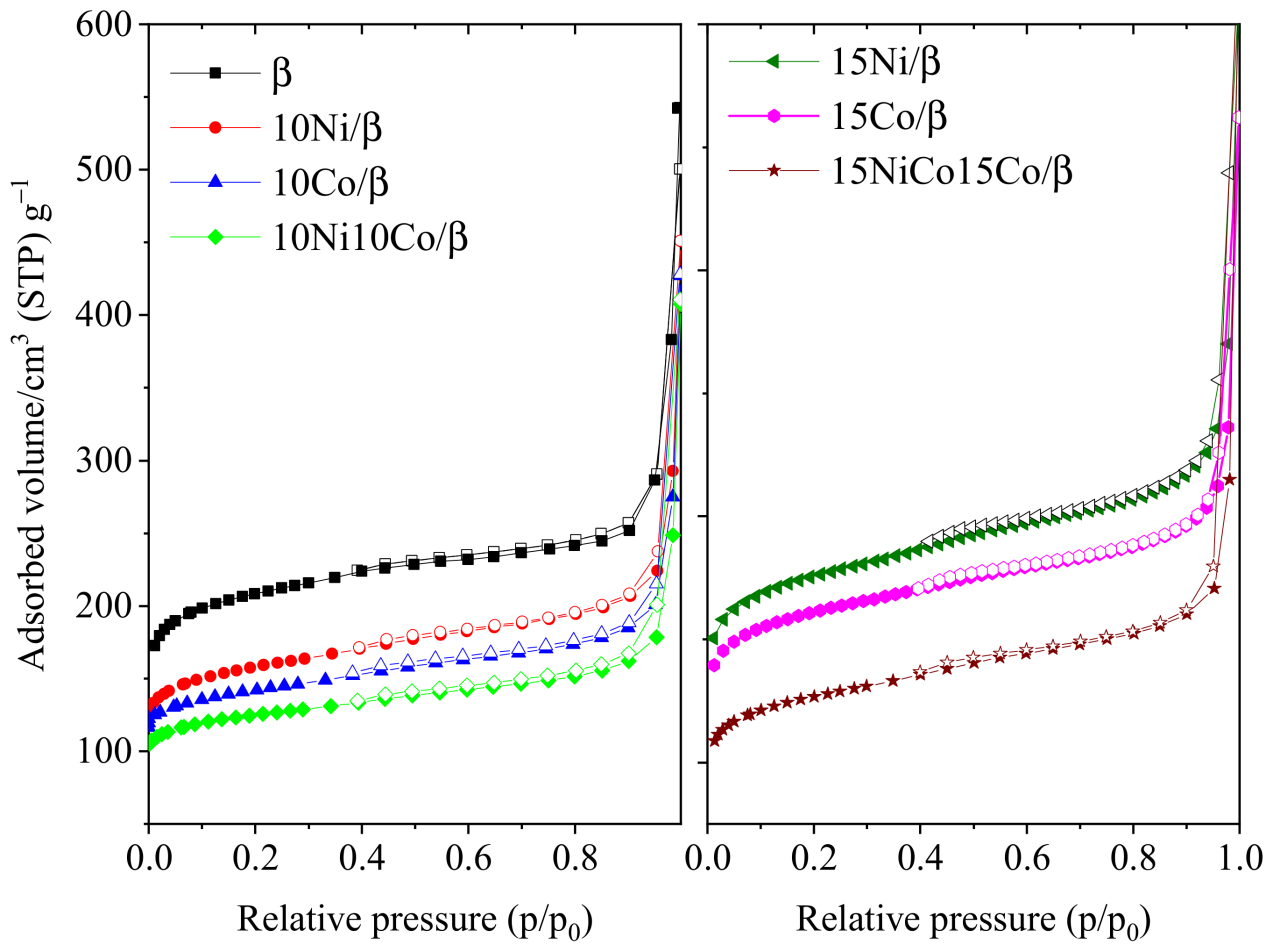
Nitrogen physisorption isotherms of the mono- and bimetallic β zeolite materials (open symbols are for the desorption branches). The isotherms of 15Co/β and 15Ni/β samples are shifted to 25 and 50 cm^3^/g higher values, respectively.

N_2_ adsorption/desorption isotherms of the parent β zeolite and its mono- and bimetallic modifications are presented in Figure 2. All isotherms are of Type I, characteristic of microporous materials. A narrow hysteresis loop observed in the relative pressure range of 0.42–0.90 indicates the presence of a small amount of mesopores with a broad pore size distribution. The steep increase in the adsorbed volume at relative pressures above 0.95 can be attributed to secondary mesoporosity arising from interparticle voids between the zeolite nanocrystals. The calculated textural parameters are summarized in Table 1.

Modification of the β zeolite with Co and/or Ni results in a decrease in both the specific surface area and the pore volume. This reduction cannot be attributed solely to the presence of non-adsorbing metal oxide species, as the micropore volume also decreases significantly in higher metal-loaded catalysts. These findings suggest that the metal ions are incorporated into the zeolite cation-exchange sites, partially blocking access to the microporous channels.

**Figure 3:**
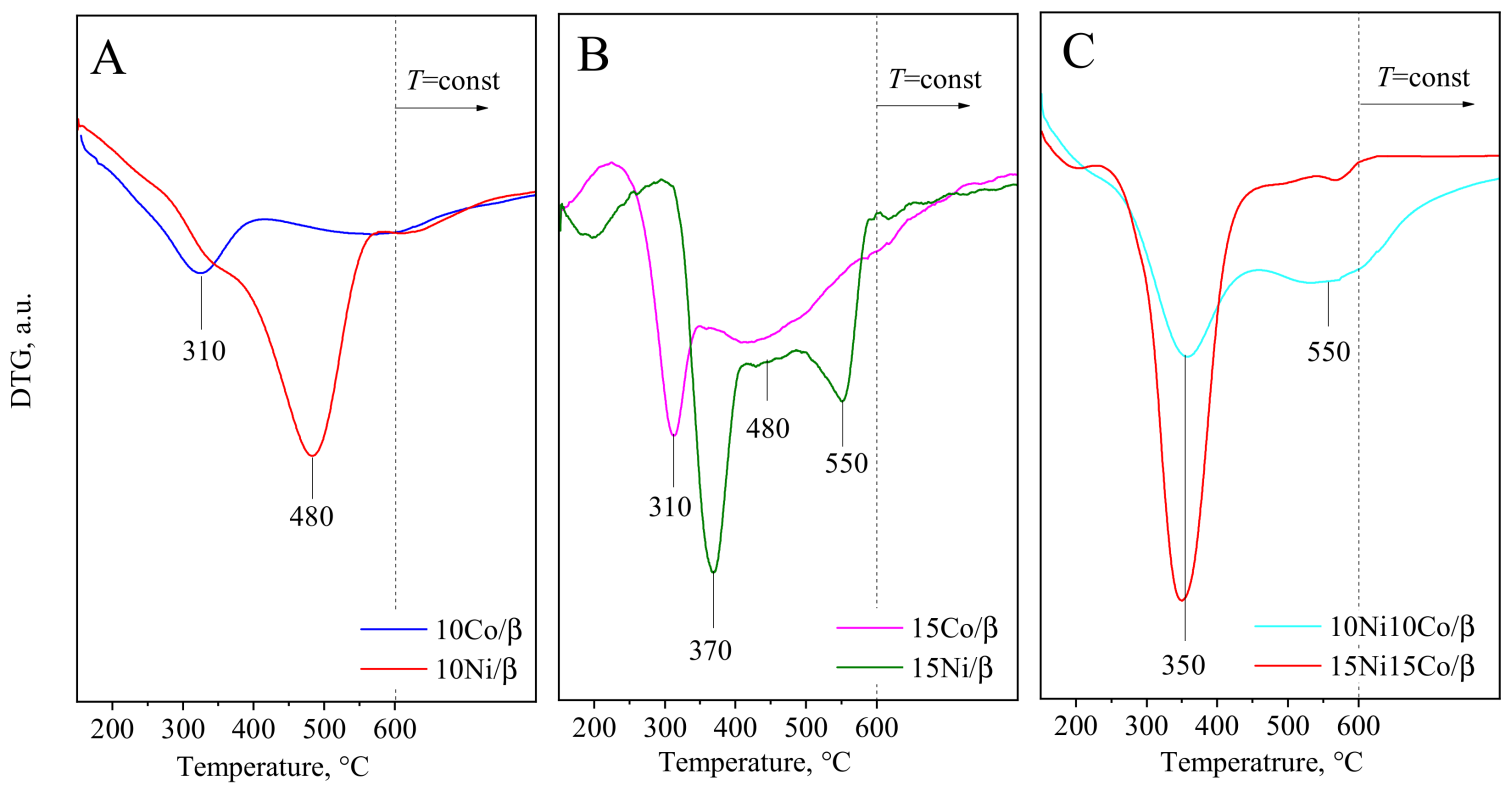
TPR-TG profiles of the monometallic 10 wt.% Ni and Co supported on β zeolite (**A**), monometallic 15 wt.% Ni and Co supported on β zeolite (**B**) and bimetallic 10Ni10Co/β and 10Ni10Co/β catalysts (**C**).

The reducibility of the mono- and bimetallic materials supported on zeolite β was investigated by temperature-programmed reduction (TPR) (Figure 3), and the reduction degrees of the metal oxide species are summarized in Table 1. The studied catalysts are characterized by a low and a higher temperature reduction region, between the 200–400 and the 400–600 °C temperature range, respectively. The more easily reducible part is associated with metal oxide nanoparticles with different sizes on the external surface of zeolite, whereas the hardly reducible species are those ones that interact with the zeolite matrix. The latter ones most probably are species incorporated into cationic positions of the zeolite, or ones reacting with the silanol groups of defect sites highly abundant in zeolite Beta. These species act as well as weak Lewis acid centers. From this point of view, cobalt is more prone to strongly interact with silicates compared to nickel. The ratio of hardly and easily reducible parts of metal oxide depends on the amount of loaded metal and the strength of interaction with the zeolite in accordance with the chemical nature of the transition metal [32,33]. These facts are mirrored in the reduction curve of 10 wt.% cobalt oxides on β zeolite, which is characterized by a low 310 °C peak and a less intensive, elongated broad peak. The low reducibility observed for the sample is most probably associated with the formation of hard-to-reduce cobalt silicate species. In contrast, the monometallic 10 wt.% Ni sample exhibits two characteristic reduction peaks at approximately 350 and 480 °C, indicating the presence of a relatively small amount of highly dispersed NiO and a larger fraction of weakly interacting, more readily reducible Ni species. The total amount of loaded Ni, corresponding to 10 wt.% NiO, can be reduced during the TPR experiment.

For the 15 wt.% Ni catalyst, the ratio between the low- and high-temperature reduction peaks changed, with the low-temperature peak becoming dominant. Nevertheless, the overall degree of reduction was slightly lower (91.5%). For the 15 wt.% Co/β catalyst, the reduction occurred within a similar temperature range; however, the degree of reduction increased significantly to 85.4%. This behavior can be attributed to the enhanced contribution of low-temperature reduction, associated with a higher proportion of separate Co_3_O_4_-derived nanoparticles.

The bimetallic Ni–Co modifications resulted in enhanced low-temperature reducibility, as evidenced by the highly intense reduction peak at 350 °C in the TPR profiles. This reduction feature suggests the formation of a mixed Ni–Co oxide spinel phase, most probably NiCo_2_O_4_, on the zeolite support in the 15Ni15Co catalyst. The formation of this phase indicates a stronger interaction between Ni and Co species than between the individual metal species and the silica–alumina support, consistent with the literature [34]. At 450 °C, the reduction treatment applied to the catalyst prior to the catalytic run is expected to reduce the metal oxides predominantly to separate Ni^0^ and Co^0^ species, although the formation of a Ni–Co solid solution cannot be excluded. An important advantage of NiCo_2_O_4_ is that the interaction between Ni and Co facilitates reduction at lower temperatures compared with isolated metal oxides that strongly interact with the support. Mixed Ni–Co oxides have been reported to exhibit lower reduction temperatures than their monometallic counterparts, supporting the possible formation of a mixed Ni–Co spinel oxide phase in our catalyst too [35].

The reduction behavior of the 10Ni10Co catalyst, however, appears to be more complex. The presence of a high-temperature reduction peak at approximately 550 °C suggests a stronger interaction between the metal species and the support, resulting in the formation of more strongly bound and therefore hardly reducible metal species.

**Figure 4:**
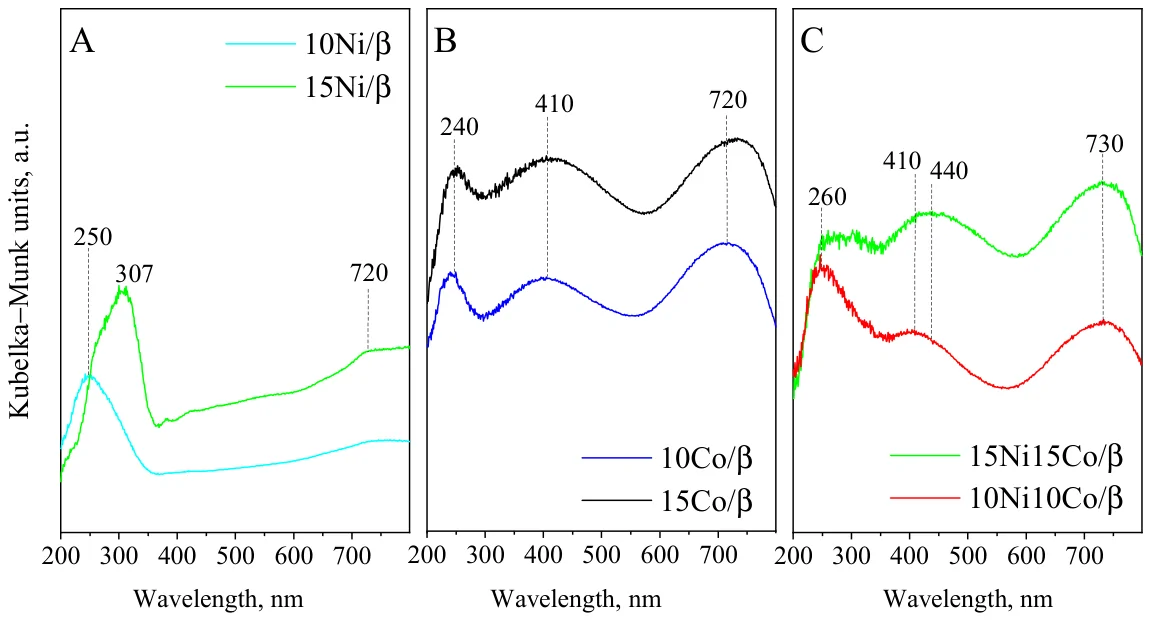
UV–Vis spectra of the monometallic Ni (10–15 wt.%) supported on β zeolite (**A**), Co (10–15 wt.%) supported on β zeolite (**B**), and bimetallic 10Ni10Co/β and 10Ni10Co/β catalysts (**C**).

Diffuse-reflectance UV–Vis (DR UV–Vis) spectra recorded at room temperature were used to characterize the nature and coordination environment of the cobalt and nickel species in the investigated catalysts (Figure 4). For 10Ni/β, a strong absorption band in the UV region at approximately 250 nm (Figure 4A) was observed, which can be attributed to ionic Ni^2+^ species associated with Ni–O–Si linkages formed during calcination, following impregnation [36]. In contrast, the spectrum of 15Ni/β exhibited a shift in the corresponding band toward approximately 307 nm, accompanied by increased intensity, as well as the appearance of new bands in the 600–750 nm region. The shift toward longer wavelengths can be associated with the formation and growth of oligomeric Ni–O species. The bands in the visible region can be attributed to the presence of a separate NiO phase, in agreement with the XRD results (Table 1). Thus, the DR UV–Vis results further support the conclusion that increasing the Ni loading promotes the formation and growth of larger, more crystalline NiO species.

The spectrum of 10Co/β (Figure 4B) exhibits three main absorption bands centered at approximately 250, 410, and 720 nm. The band in the UV region can be associated with ligand-to-metal charge-transfer transitions involving ionic Co species, most probably interacting with the silica–alumina support. The bands at approximately 410 and 720 nm can be assigned to electronic transitions involving Co^2+^ and Co^3+^ species in a spinel structure [34]. The presence of these characteristic bands supports the formation of Co_3_O_4_ species. For 15Co/β, the same absorption features were observed, but with increased intensity, particularly in the visible region. This behavior is consistent with the higher cobalt loading and the formation of a larger fraction of Co_3_O_4_ species, in agreement with the XRD results (Table 1).

The spectra of the Ni–Co bimetallic catalysts show three broad absorption bands centered at approximately 260, 410–440, and 720–740 nm. The band at around 260 nm can be associated with Ni^2+^ species, whereas the other two bands are characteristic of electronic transitions involving Co^2+^ and Co^3+^ species in Co_3_O_4_. The presence of these bands indicates the coexistence of Co^2+^ and Co^3+^ species and can be explained by the formation of cobalt oxide with a spinel structure. The bands at 410–440 and 720–740 nm are more intense for 15Ni15Co/β, consistent with its higher content of crystalline Co_3_O_4_ species. The higher intensity of these bands compared with those of 10Ni10Co/β reflect the XRD results, which indicate a greater contribution of crystalline Co_3_O_4_ at the higher total metal loading.

The intensity of 260 nm band in 10Ni10Co/β catalyst is higher compared to that of 15Ni15Co, showing higher fraction of ionic species interacting with the support as was also observed by the TPR method. The low wavelength band is shifted toward 260–320 nm in 15Ni15Co/β. This red shift can be attributed to the formation of larger NiO particles at the higher Ni loading, in agreement with the XRD results, indicating 11 and 14 nm sized NiO crystallites for 10Ni10Co/β and 15Ni15Co/β, respectively.

The combined DR UV–Vis and XRD results suggest that higher metal loading favors the formation of larger NiO and Co_3_O_4_ domains, whereas at lower loading a greater fraction of highly dispersed, support-interacting metal species are present.

The parent zeolite β and the Ni/Co-modified catalysts were characterized by quantitative ^29^Si and ^27^Al solid-state NMR spectroscopy to assess structural changes induced by metal impregnation. The ^29^Si spectra were deconvoluted using DMFit v. 20230120 software [37] to identify the contributions of the different Si environments, and the results are summarized in Table 2.

The Si spectrum of the parent zeolite β exhibits four main resonances centered at around −114, −110, −105 and −102 ppm, together with a low-intensity resonance at around −98 ppm (Figure S1). The signals at −114 and −110 ppm are assigned to crystallographically nonequivalent Q4 species (Si(0Al)), whereas the signal at −105 ppm corresponds to Si atoms bonded to one Al atom (Si(1Al)). The signal at −102 ppm can be attributed to Si(2Al) and/or silanol groups (Si(1OH)). The presence of defect silanol sites was further supported by ^1^H-^29^Si cross-polarization (CPMAS) experiments (Figure S2), which showed enhanced intensities of the resonances at −102 and −98 ppm, confirming the presence of Si(1OH) and a minor amount of Si(2OH) species, respectively.

**Figure 5:**
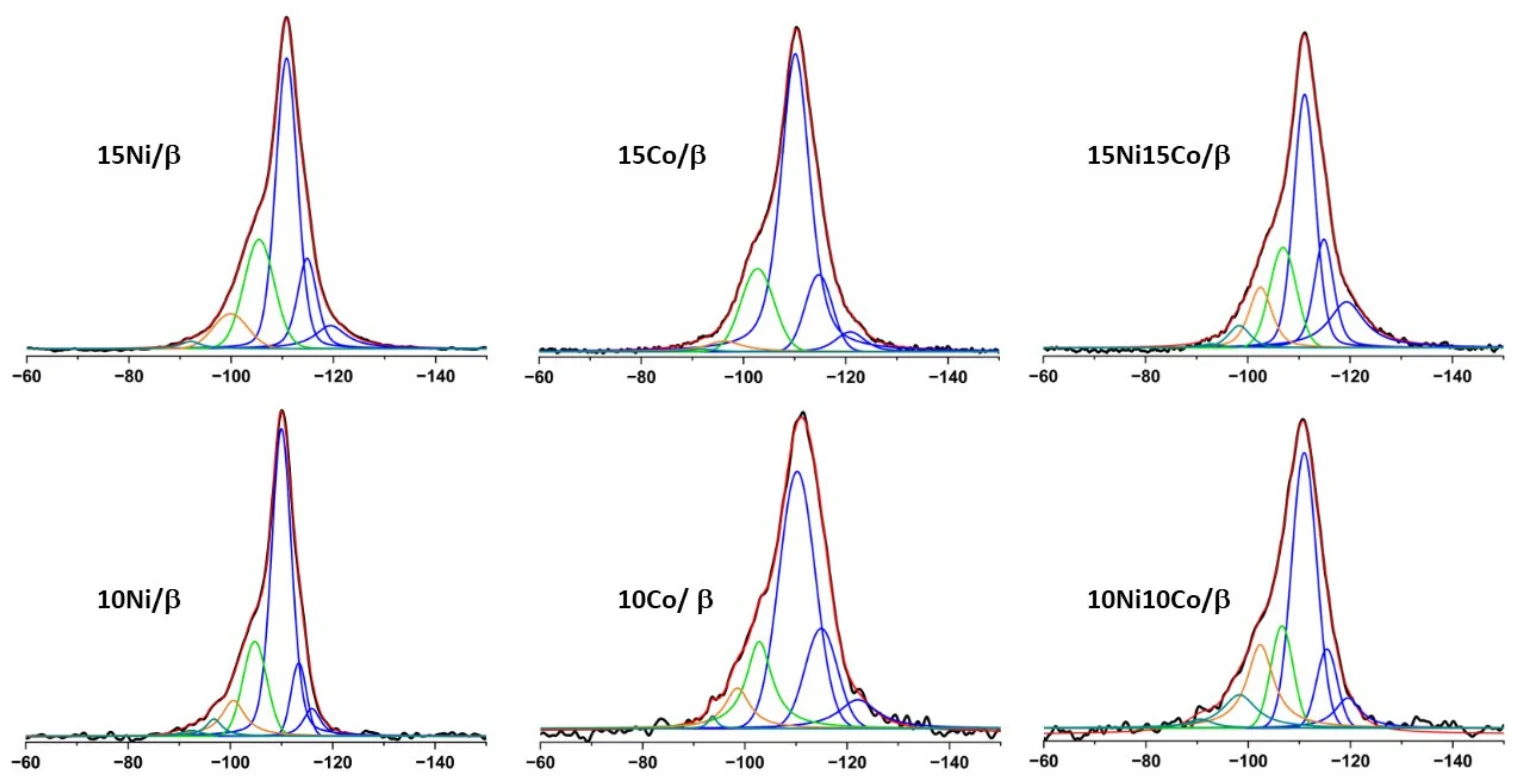
^29^Si NMR spectra of metal-modified catalysts with the results of the deconvolution. A black line represents the experimental spectrum, a red line the overall fit; the individual components are presented with different colors: Si(0Al)—blue; Si(1Al)—green; Si(2Al); Si(1OH)—light brown; minor components—dark green.

Metal impregnation resulted in noticeable changes in the ^29^Si spectra, indicating increased structural and compositional heterogeneity of the zeolite matrix of all metal-modified catalysts compared with parent zeolite β. In all metal-modified samples, an additional high-field resonance appeared in the range of −116 to −122 ppm (Figure 5). Since Q4 species in zeolites typically resonate within the −110 to −115 ppm range, this additional contribution suggests a perturbation of the local Si–O framework environment, potentially associated with the incorporation or dispersion of metal oxide species within the zeolite structure. Such interactions may induce distortions of Si–O bond lengths and O–Si–O/Si–O–Si bond angles in affected Q4 units. Furthermore, the two bimetallic catalysts and the 10Ni/β sample exhibited an additional low-field contribution at approximately −91 to −92 ppm. This feature may be associated with defect Si sites resulting from interactions with surface oxide species.

**Figure 6:**
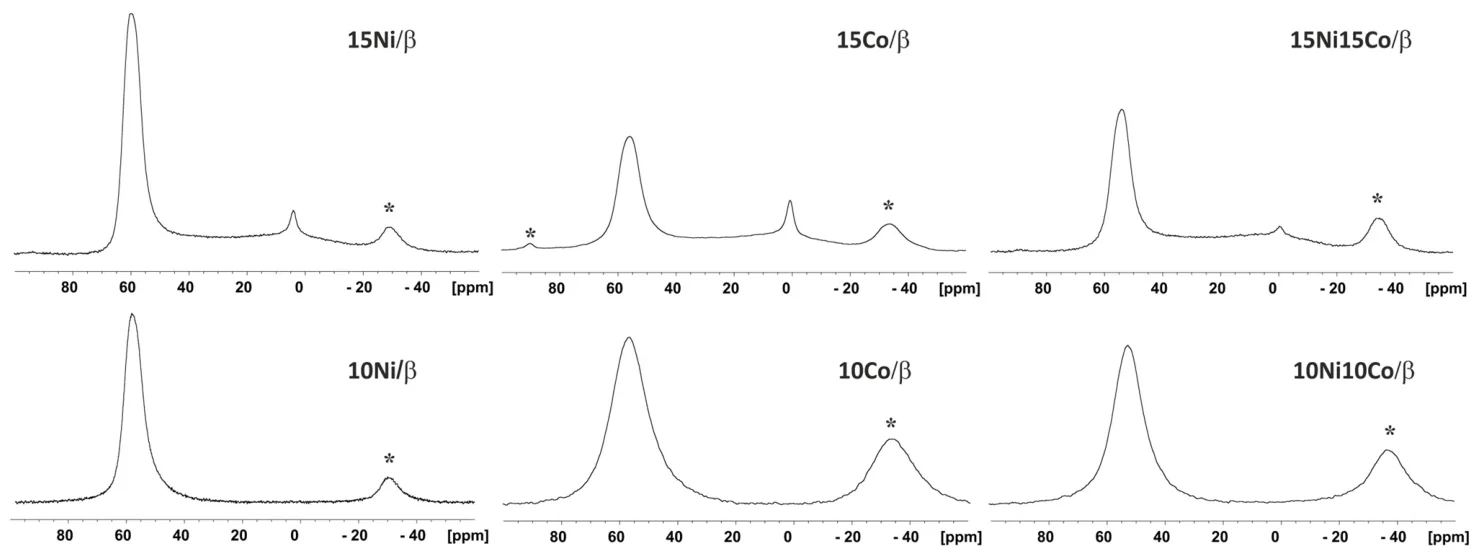
^27^Al NMR spectra of metal-modified catalysts. * denotes spinning side bands.

Complementary information on the state of Al was obtained from the ^27^Al NMR spectra (Figure 6). The spectrum of the parent zeolite is dominated by two partially overlapping resonances centered at approximately 60 ppm, characteristic of tetrahedrally coordinated framework Al species (FAl) with different local symmetries (Figure S3). Additional narrow and broad resonances near 0 ppm indicate the presence of various extra-framework Al (EFAl) species. Interestingly, impregnation with 10% Ni and/or 10% Co resulted in the disappearance of the EFAl signals from the ^27^Al spectra of all modified materials.

At higher metal loadings, however, more pronounced changes in the ^27^Al spectra were observed in both mono- or bimetallic catalysts (15Ni/β, 15Co/β and 15Ni15Co/β). In all three samples, alongside the dominant resonance associated with tetrahedral framework Al, a noticeable contribution from EFAl species appeared as a sharp resonance near 0 ppm, accompanied by a broad component extending from approximately −20 to +45 ppm. The latter resonance is typically attributed to defect framework Al^V^ sites with low symmetry resulting in significant quadrupolar broadening of the Al resonance. Together with the changes observed in the ^29^Si spectra, these results indicate that high metal loadings induce significant modifications of the local Si and Al environments. The increased contribution of EFAl species further suggests partial disruption of the zeolite framework and possible dealumination under these preparation conditions, resulting in a more heterogeneous distribution of framework and extra-framework species.

In addition to the above discussed changes, the introduction of metal species resulted in line broadening of the main tetrahedral Al resonance at approximately 60 ppm in all samples. However, the signal broadening did not monotonically increase with metal loading but showed the following trend: parent zeolite β < 15Ni/β < 10Ni/β~15Ni15Co/β < 15Co/β < 10Ni10Co/β < 10Co/β. Therefore, a narrower linewidth was observed for the samples with 15% metal loading compared with the corresponding resonance in the 10% sample. Although an increase in linewidth might generally be expected with increasing concentration of paramagnetic metal species, the linewidth is governed not only by the total metal content but also by the spatial distribution and local environment of the paramagnetic centers relative to the Al sites. The narrower resonance at higher metal loading may therefore indicate a redistribution or partial aggregation of metal oxide species, resulting in a lower fraction of framework Al sites being in close proximity to paramagnetic metal centers. Alternatively, the higher metal loading may induce a reorganization of framework Al environments, producing a more homogeneous population of tetrahedral Al sites and consequently a narrower resonance. Thus, the non-monotonic change in linewidth suggests that the local distribution of metal oxide species, rather than their overall concentration alone, plays an important role in determining the ^27^Al NMR response.

**Figure 7:**
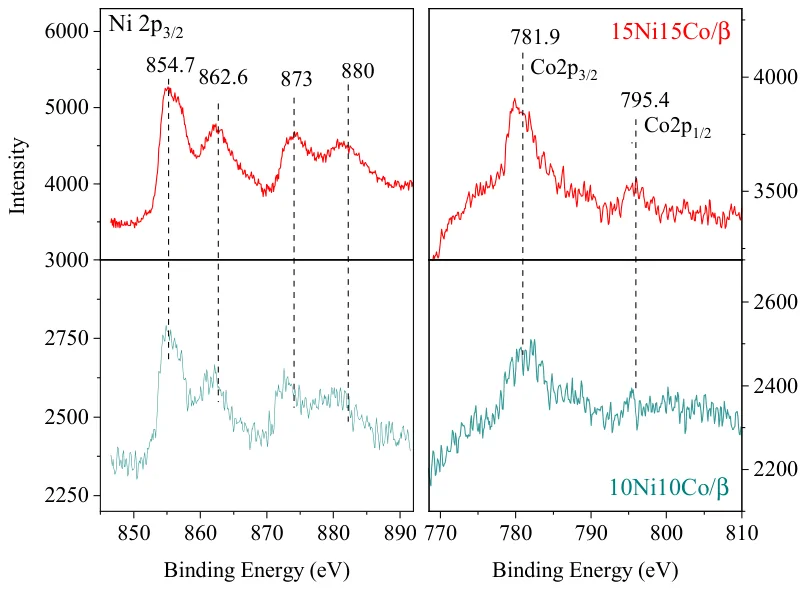
Ni and Co XPS spectra of 10Ni10Co/β and 15Ni15Co/β catalysts.

XPS spectra of the bimetallic 10Ni10Co/β and 15Ni15Co/β catalysts were used to investigate the nature and surface concentration of the metallic species deposited on the zeolite surface, owing to their importance in the catalytic process (Figure 7). The Ni 2p XPS spectra of both samples exhibit a Ni 2p_3/2_ peak at approximately 854.7 eV, accompanied by characteristic satellite features at 862.6, 873, and 880 eV, which are characteristic of Ni^2+^ species [38,39,40]. The Co 2p spectra show features at binding energies of approximately 781.9 and 795.4 eV, assigned to the Co 2p_3_/_2_ and Co 2p_1_/_2_ regions, respectively. These features are consistent with the presence of oxidized cobalt species, although the overlapping signals do not allow the contributions of Co^2+^ and Co^3+^ to be distinguished unambiguously. The similar binding energies and spectral features of the two catalysts suggest that increasing the metal loading does not substantially alter the chemical states of the surface Ni and Co species. However, the higher intensity of the Co 2p_3_/_2_ feature at approximately 781.9 eV for 15Ni15Co/β may indicate an increased surface contribution from Co^2+^ species. This difference may reflect variations in the relative abundance and surface distribution of cobalt species; however, the available XPS data do not provide sufficient evidence to confirm the formation of a specific mixed Ni–Co oxide phase.

Nickel was predominantly exposed on the zeolite surface in both samples, with surface concentrations of 3.7 and 13.4 at.% for 10Ni10Co/β and 15Ni15Co/β, respectively. In contrast, only a small fraction of cobalt was detected at the catalyst surface, with surface concentrations of 0.9 and 1.5 at.% for 10Ni10Co/β and 15Ni15Co/β, respectively. The substantially higher surface Ni concentration in 15Ni15Co/β compared with 10Ni10Co/β indicates a greater abundance of Ni species accessible at the outer surface of the zeolite (Table 3). These results are consistent with the H_2_-TPR data, which show facilitated reduction of Ni species in the bimetallic catalysts. This behavior may be associated with the higher density of Ni nanoparticles at the catalyst surface and possible interactions between Ni and Co species, which facilitate the reduction of Ni-containing species.

**Figure 8:**
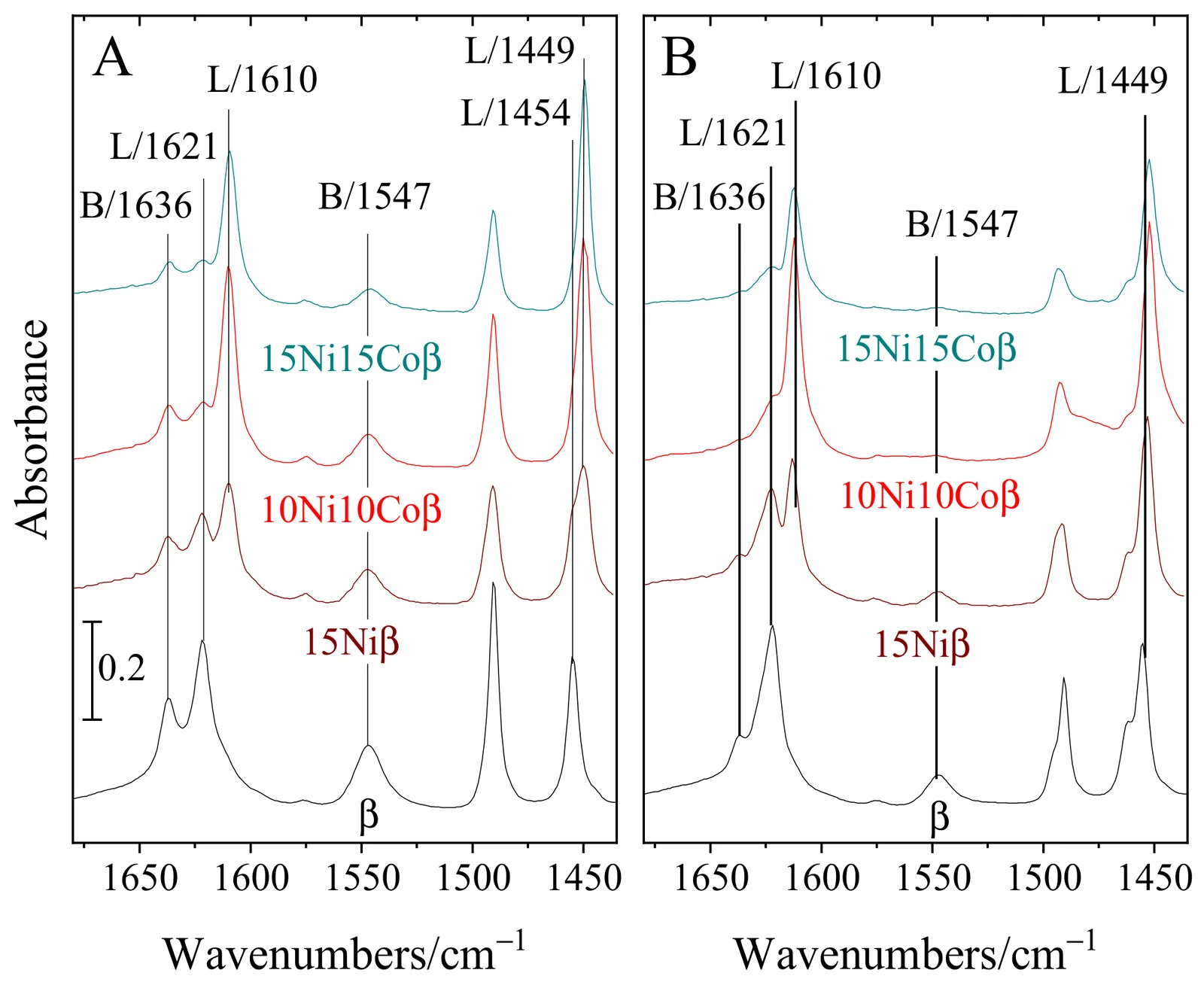
FT-IR spectra of adsorbed pyridine on β and metal-modified β zeolites. Samples were dehydrated at 350 °C, then contacted with 5 mbar pyridine vapor at 100 °C, followed by evacuation at 200 (**A**) and 400 °C (**B**). Spectra are normalized to 5 mg/cm^2^ thickness of pellet.

The acidity of the catalysts was characterized by FT-IR spectroscopy of adsorbed pyridine (Py), as shown in Figure 8.

Pyridine protonated at Brønsted acid sites (B) gives rise to a pair of bands at 1545 and 1635 cm^−1^, corresponding to the 19b and 8a ring vibration modes, respectively [41]. Lewis acid sites (L) are identified by Py adsorption bands in the 1440–1455 and 1610–1620 cm^−1^ regions. The parent zeolite β exhibits bands characteristic of both Brønsted and Lewis acid sites. The Lewis acidity of zeolites generally originates from extra-framework alumina species (AlO^+^); however, framework tri-coordinated Al^3+^ defect sites in zeolite β can also act as Lewis acid centers [42]. The latter species give rise to bands at 1454 and 1621 cm^−1^.

Modification of the zeolite with transition metals resulted in the incorporation of metal cations into cationic positions within the zeolite framework, as indicated by the appearance of two new bands at 1449 and 1610 cm^−1^. However, Ni and Co ions may also coordinate to framework Al defect sites, thereby eliminating the coordinative unsaturation of the parent zeolite β. With increasing metal loading, the intensities of the bands at 1547 cm^−1^ (B) and 1455 cm^−1^ (L) decrease proportionally, suggesting that a substantial proportion of the framework protons and defect sites become occupied by Ni or Co cations.

The strength of the acid sites can be assessed based on the desorption temperature of Py. Figure 8B shows the spectra recorded after Py desorption at 400 °C. The results indicate that both the number and strength of the Brønsted acid sites decrease with increasing metal content, as evidenced by the negligible amount of Py remaining adsorbed on the Brønsted acid sites of the 10Ni10Co/β and 15Ni15Co/β catalysts compared with the parent zeolite β after high-temperature desorption (Table S1). These findings suggest that Ni and Co ions preferentially interact with the strongest acid sites, which may significantly affect the dehydration activity of the catalysts.

The catalytic activity of the mono- and bimetallic zeolite catalysts for the hydrogenation of levulinic acid (LA) to γ-valerolactone (GVL) was evaluated at 150 °C, and the results are summarized in Table 2. The main products identified were γ-valerolactone (GVL) and 4-hydroxypentanoic acid (4-HPA), the latter being an intermediate in the conversion of LA to GVL through subsequent cyclization. The monometallic Co-containing catalysts exhibited low LA conversion, ranging from 2% LA conversion for the catalyst containing 10 wt.% Co to 10% conversion for the one containing 15 wt.% Co. In contrast, the Ni-containing catalysts showed higher activity, with LA conversions of 12.6% and 24.8% for the catalysts containing 10 wt.% and 15 wt.% Ni, respectively. These results suggest that catalytic performance depends on both metal nature and its loading, with the Ni-containing catalysts exhibiting higher activity than their Co-containing counterparts under the investigated reaction conditions.

In contrast, a pronounced enhancement in catalytic activity was observed for the bimetallic catalysts. The LA conversion increased from 53 wt.% for 10Ni10Co/β to complete conversion (100 wt.%) for 15Ni15Co/β. This increase in activity was accompanied by a corresponding increase in GVL yield, from 48 wt.% to 92 wt.%, respectively. This improvement can be attributed to the synergistic interaction between Ni and Co species, which may facilitate hydrogen activation and promote the hydrogenation of the carbonyl group of levulinic acid. The higher total metal loading in 15Ni15Co/β likely also resulted in a greater number of accessible active sites, contributing to the substantially higher LA conversion.

**Scheme 1:**
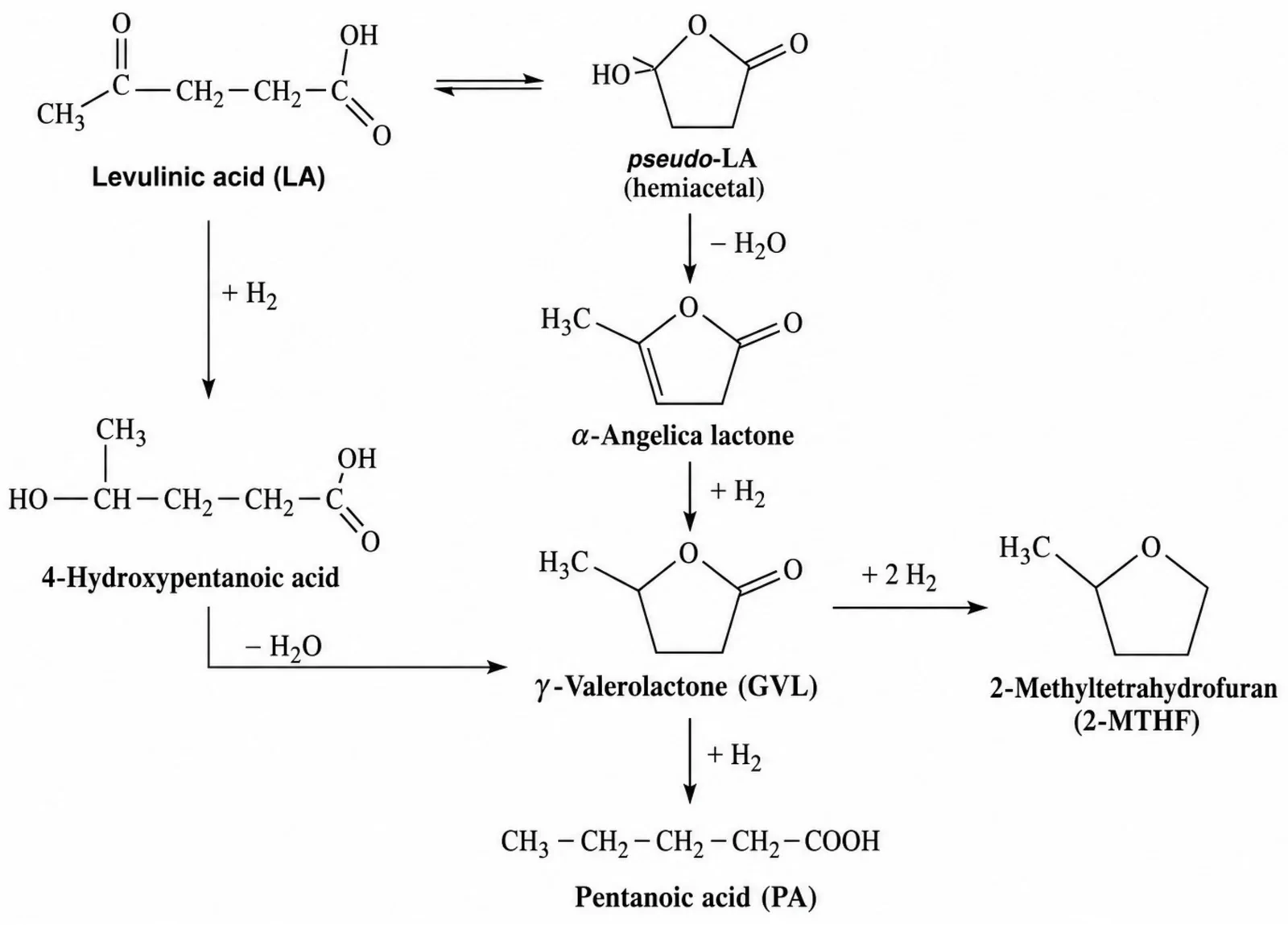
Reaction pathways of levulinic acid hydrogenation.

Regarding product distribution, γ-valerolactone was the predominant product for both bimetallic catalysts. Only a relatively small amount of 4-hydroxypentanoic acid was detected, with yields of 5 and 8 wt.%, respectively. This suggests that the hydrogenation step is followed by rapid intramolecular esterification/cyclization under the applied reaction conditions. The formation of 4-HPA as a minor product is consistent with its role as an intermediate in the conversion of LA to GVL in accordance with pathway II, Scheme 1. Thus, the high GVL yield obtained over the bimetallic catalysts, particularly 15Ni15Co/β, demonstrates the effectiveness of the Ni–Co active sites in promoting the desired reaction pathway (Scheme 1).

The cyclic experiments revealed a pronounced stability in the catalytic performance of the bimetallic Ni–Co/β-zeolite catalysts compared with their monometallic counterparts. As indicated in Table 4, the conversion values obtained over three reaction cycles (shown in parentheses) remained relatively low for the monometallic catalysts, reaching 1.0% for 10Co/β, 5.6% for 10Ni/β, 3.8% for 15Co/β, and 10.0% for 15Ni/β. In contrast, the bimetallic catalysts exhibited substantially higher conversions of 50.0% for 10Ni10Co/β and 98.0% for 15Ni15Co/β. The latter catalyst achieved complete LA conversion (100%) in the initial experiment, with a GVL yield of 92 wt.% and a 4-HPA yield of 8 wt.%, while maintaining nearly complete conversion over the three reaction cycles. Similarly, 10Ni10Co/β exhibited a conversion of 53%, with GVL and 4-HPA yields of 48 and 5 wt.%, respectively, although its conversion decreased to 50% in the cyclic experiments. These results demonstrate the superior catalytic performance of the bimetallic catalysts, particularly 15Ni15Co/β, and suggest a beneficial synergistic effect between Ni and Co. The small decrease in conversion over repeated cycles indicates relatively stable catalytic performance under the investigated conditions.

The hydrogen chemisorption results (Table S2) reveal differences in the dispersion and accessibility of the metal species among the studied catalysts. For the monometallic Ni/β catalysts, increasing the nominal Ni loading from 10 to 15 wt.% increases the hydrogen uptake from 50.58 to 70.02 µmol g^−1^, while the Ni dispersion decreases slightly from 5.94 to 5.48%. The corresponding metal surface area per gram of catalyst increases from 3.96 to 5.48 m^2^ g^−1^, whereas the estimated Ni particle size increases from 17.0 to 18.4 nm. These results indicate that the higher Ni loading increases the total amount of accessible Ni, despite a slight decrease in dispersion. A similar trend is observed for the monometallic Co/β catalysts: increasing the Co loading increases the hydrogen uptake from 13.80 to 32.04 µmol g^−1^ and the metal surface area from 1.10 to 2.56 m^2^ g^−1^, accompanied by an increase in Co dispersion from 1.63 to 2.52% and a decrease in the estimated particle size from 61.2 to 39.6 nm.

The bimetallic catalysts exhibit distinct chemisorption and catalytic behavior. The 10Ni10Co/β sample shows the highest dispersion (7.06%) and the smallest estimated particle size (14.3 nm) among the catalysts listed. Its hydrogen uptake reaches 60.12 µmol g^−1^, and its metal surface area is 4.70 m^2^ g^−1^ of catalyst. Importantly, this catalyst exhibits a TOF of 0.02636 s^−1^, considerably higher than those of the monometallic Ni/β and Co/β catalysts, for which the TOF values range from 0.00650 to 0.01059 s^−1^. This enhancement suggests that the combination of Ni and Co may improve the catalytic effectiveness of the accessible metal sites.

The 15Ni15Co/β catalyst exhibits the highest hydrogen uptake (72.57 µmol g^−1^) and the largest metal surface area per gram of catalyst (5.68 m^2^ g^−1^) among the samples studied. Its estimated particle size is 17.8 nm, while its dispersion (5.68%) is lower than that of 10Ni10Co/β. Despite this lower dispersion and larger particle size, 15Ni15Co/β achieves a TOF of 0.04120 s^−1^, approximately 56% higher than that of 10Ni10Co/β. This result indicates that catalytic performance is not governed exclusively by metal dispersion or particle size. Differences in the chemical nature of the accessible sites, the interaction between Ni and Co, and the metal–support interface may also contribute to the observed activity.

**Figure 9:**
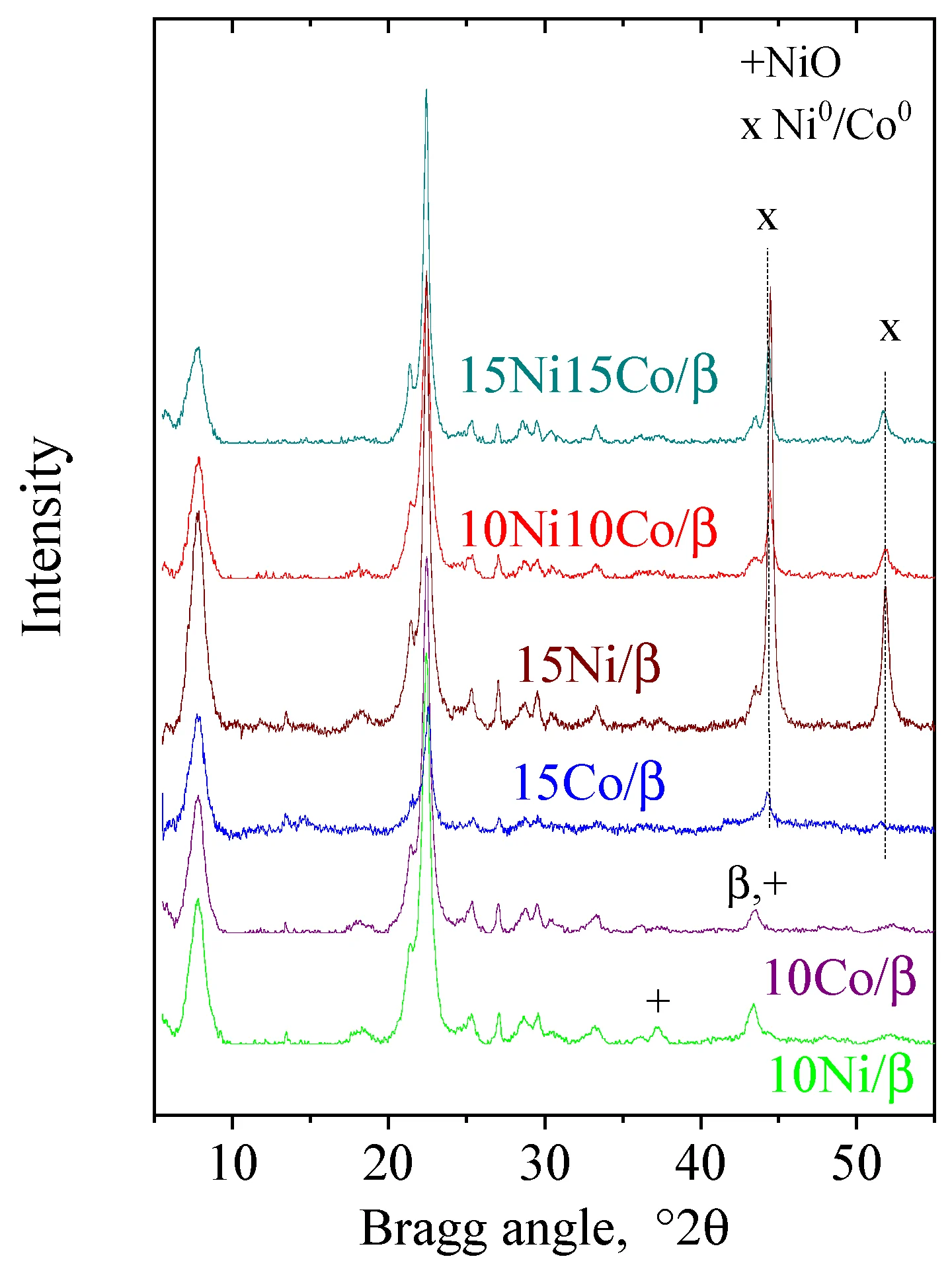
X-ray powder diffraction patterns of the spent metal-modified β zeolites.

XRD analysis of the spent catalysts was performed to investigate the formation and structural characteristics of the metallic phases generated during the reaction, which may represent the active sites responsible for the catalytic performance (Figure 9).

The patterns revealed the presence of crystalline metallic Co^0^/Ni^0^ phases in both bimetallic catalysts. The reflections can be attributed to the Co^0^, Ni^0^, and/or Co–Ni alloy phases; however, due to the similar unit cell sizes of cubic systems (Ni^0^–a_0_ = 3.524 Å, Co^0^–a_0_ = 3.545 Å), the identification and individual contribution of each phase to the calculated crystallite size cannot be determined unambiguously from XRD. For 10Ni10Co/β and 15Ni15Co/β, the detected metallic nanoparticles had an average crystallite size of approximately 35 nm. In the case of the monometallic 15Ni/β catalyst, bigger metal particles with sizes of 43 were detected.

The XRF data (Table S3) indicate some changes in the chemical composition of the catalysts before and after the catalytic reaction. A more pronounced change in the metal content is observed for cobalt before and after the catalytic experiment in both the monometallic and bimetallic catalysts. For 15Co/β, the Co concentration decreases from 16.47 to 7.76 wt.% after the reaction, and for 10Ni10Co/β a similar decrease in Co content from 9.36 to 3.76 wt.% is observed. In contrast, the Ni concentration changes to a much smaller extent, decreasing from 8.72 to 7.14 wt.% for 10Ni10Co/β and from 11.87 to 10.49 wt.% for 15Ni15Co/β. Moreover, the Ni concentration in the monometallic 15Ni/β sample is essentially preserved after the reaction, changing only slightly from 16.53 to 17.26 wt.%. The relatively small changes in Ni content, particularly in the Ni-rich catalysts, indicate a comparatively high stability of the Ni species under the applied reaction conditions. The relatively high Ni content retained in 15Ni15Co/β, together with its highest hydrogen uptake (Figure S4), suggests that this catalyst preserves a substantial concentration of hydrogen-accessible metal sites after reaction. Overall, the XRF results indicate that Ni is comparatively stable under the applied reaction conditions, whereas the Co-containing catalysts exhibit more pronounced changes in their apparent Co content, particularly for the monometallic 15Co/β and lower-loaded bimetallic 10Ni10Co/β catalysts.

**Figure 10:**
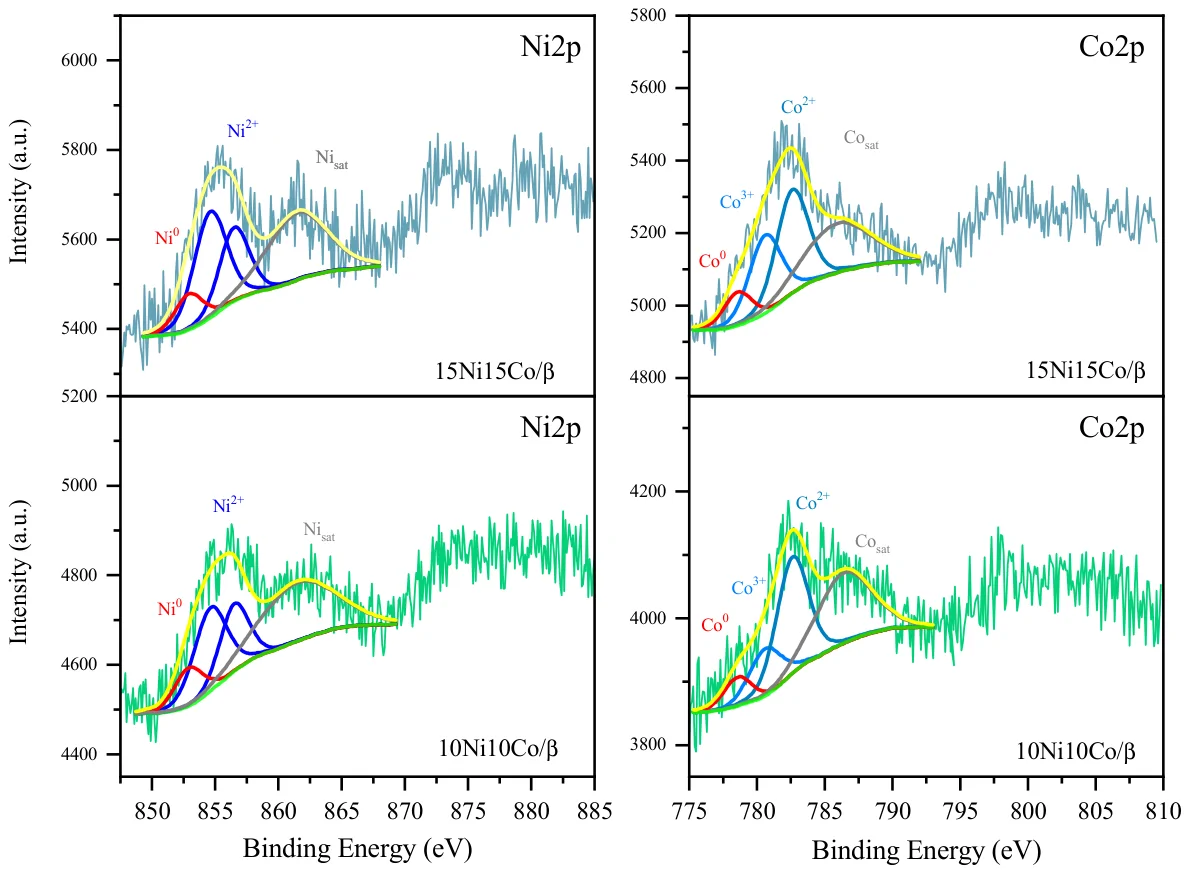
Ni and Co XPS spectra of the spent 10Ni10Co/β and spent 15Ni15Co/β catalysts.

The XPS spectra of the spent bimetallic catalysts reveal the coexistence of metallic Ni and Co species with oxidized Ni^2+^ and Co^2+^/Co^3+^ species, which may result from the rapid reoxidation of the metal particles upon exposure to air after the catalytic reaction (Figure 10). The slight shift in the Ni 2p_3_/_2_ peak from 852.9 eV for 10Ni10Co/β to 853.1 eV for 15Ni15Co/β suggests a subtle modification of the electronic environment of Ni with increasing Co content. This shift may be associated with electronic interactions between Ni and Co and could be consistent with the formation of Ni–Co alloy species, although the peak shift alone is insufficient to confirm alloy formation.

Interestingly, the higher Ni and Co loading in the 15Ni15Co/β catalyst resulted in the formation of smaller metallic crystallites compared with 10Ni10Co/β. This observation suggests that the higher metal loading may promote the formation of a larger number of smaller and more highly dispersed metallic particles, rather than extensive particle growth, in connection with the interaction of metallic nanoparticles and the zeolite support. In addition to the metallic phases, a fraction of unreduced NiO and/or reoxidized Ni species, estimated at approximately 7–15%, was also detected in the spent bimetallic catalysts. The presence of both metallic and oxidized Ni species indicates that partial oxidation of the Ni phase may have occurred during or after the catalytic reaction.

The higher proportion of finely dispersed metallic Co^0^/Ni^0^ nanoparticles in 15Ni15Co/β may contribute to its superior catalytic performance. In particular, the smaller crystallite size and potentially greater number of accessible Ni–Co interfacial sites may facilitate H_2_ activation and the subsequent hydrogenation of levulinic acid, resulting in the complete conversion of LA observed for this catalyst. Therefore, the XRD results support the proposed relationship between the formation of finely dispersed bimetallic active phases and the enhanced catalytic activity of 15Ni15Co/β.

The catalytic performance of 15Ni15Co/β was compared with that of representative noble- and non-noble-metal catalysts reported for the hydrogenation of levulinic acid (LA) to γ-valerolactone (GVL) (Table S4). As summarized in Table S4, catalysts in the literature exhibit a wide range of catalytic performance, depending on the active metal, support, reaction conditions, and operating mode [27,28,43,44,45,46,47]. Among the noble-metal catalysts, the Ru-based system combined with an acidic co-catalyst achieved a GVL yield of 99 mol% at 70 °C and 0.5 MPa H_2_ after 3 h, with a reported activity of 558 h^−1^ [27]. This result demonstrates that high GVL yields can be obtained under relatively mild conditions through the combination of metal-catalyzed hydrogenation and acid-catalyzed transformations. The 5% Pt/carbon catalyst also exhibited high performance, achieving 100% LA conversion and 98.6% GVL selectivity at 265 °C and atmospheric hydrogen pressure [44]. Although both systems are highly effective, they rely on noble metals, which can increase catalyst cost and affect the economic feasibility of large-scale applications.

The non-noble-metal catalysts show similarly promising results, although their performance varies considerably. The Co/Al_2_O_3_ catalyst achieved complete LA conversion and greater than 99% GVL selectivity at 180 °C and 5 MPa H_2_ [28]. In contrast, the Ni-ZSM-5 catalyst exhibited 70.4% conversion and 61.8% GVL selectivity at 300 °C [43], while the Ni_1_Cu_1_/SiO_2_ catalyst achieved 80% conversion and 90.3% GVL selectivity at 275 °C [45]. These differences illustrate that the use of non-noble metals alone does not ensure high catalytic performance. Based on the obtained catalytic results, the nature of the metal species, their dispersion, the properties of the support, and the reaction conditions are also important. Notably, complete LA conversion and 100% GVL selectivity have also been reported for Ni_5_Mo_1_-MOF at 160 °C and 2 MPa H_2_ [46], and for Ni–Co/γ-Al_2_O_3_ at 250 °C and atmospheric pressure under vapor-phase conditions [47]. These findings indicate that appropriately designed bimetallic catalysts can be highly effective for LA hydrogenation. The performance of 15Ni15Co/β is consistent with this trend, as the catalyst achieved 100% LA conversion and 92% GVL selectivity at lower temperature. Its performance is particularly interesting because it combines non-noble-metals such as Ni and Co with a β-zeolite support, which may provide a favorable combination of metal sites for hydrogenation and support-associated acid sites.

## 3. Experimental Part

### 3.1. Materials

Sodium hydroxide (NaOH, Sigma, St. Louis, MO, USA), aluminum isopropoxide (Al(O-CH-(CH_3_)_2_)_3_, Sigma) and tetraethylammonium hydroxide (TEAOH, 20 wt.% aqueous solution, Sigma) were used for the synthesis of zeolite β. All reagents were used directly as supplied, without further purification.

### 3.2. Synthesis of Nanosized β Zeolite

Nanosized zeolite β was synthesized following the procedure reported by Landau et al. [48], with the silica precursor obtained from rice husks according to the method described in [49]. The starting synthesis mixture had the molar composition 25.00SiO_2_:0.25Al_2_O_3_:9.0R:0.35Na_2_O:295H_2_O, where R denotes tetraethylammonium hydroxide (TEAOH), which served as the organic structure-directing agent.

The precursor gel was subjected to hydrothermal crystallization at 100 °C for 9 days. After crystallization, the solid product was separated and repeatedly washed and centrifuged using deionized water until the suspension reached approximately neutral pH (pH 7). The washed material was subsequently freeze-dried. The organic template was removed by calcination in static air at 550 °C for 6 h, yielding the H-form of zeolite β.

According to ICP-OES analysis, performed after microwave-assisted digestion of the sample in an HF/HNO_3_ mixture, the calcined material contained 0.97 mmol Al/g and 0.07 mmol Na/g. The water content was 5.5 wt.%, while the bulk Si/Al ratio was 16.3. The ion-exchange capacity, determined from the temperature-programmed evolution of ammonia from the NH_4_-form of the zeolite, was 0.83 mmol/g. The resulting material was designated as β.

### 3.3. Impregnation with Ni and Co Nanoparticles of Nanosized β Zeolite

Nickel- and cobalt-containing mono- and bimetallic zeolite catalysts were prepared by impregnation of **β** zeolite using the corresponding nitrate precursors. The targeted metal loadings were 10–15 wt.% Ni and 10–15 wt.% Co. Before impregnation, the zeolite support was dehydrated at 160 °C for 2 h.

For the Ni-containing materials, 550.5 mg or 874.4 mg of Ni(NO_3_)_2_·6H_2_O was dissolved in 5 mL of ethanol. Each solution was gradually contacted with 1 g of zeolite **β** under continuous stirring, and stirring was continued until complete solvent evaporation. The resulting solids were dried at 80 °C for 18 h and subsequently calcined in air at 450 °C for 3 h using a heating rate of 1 °C/min. The products were designated **10Ni/β** and **15Ni/β**, respectively.

The Co-containing catalysts were prepared analogously. In this case, 548.7 mg or 871.7 mg of Co(NO_3_)_2_·6H_2_O was dissolved in 5 mL of ethanol and introduced into 1 g of zeolite b. After removal of the solvent under stirring, the samples were dried at 80 °C for 18 h and calcined at 450 °C for 3 h with a heating rate of 1 °C/min. The resulting samples were designated **10Co/β** and **15Co/β**, respectively.

For the bimetallic catalysts, 550.5 or 874.4 mg of Ni(NO_3_)_2_·6H_2_O was dissolved in 5 mL of ethanol and added to 1 g of the previously prepared **10Co/β** and **15Co/β** materials, respectively. After impregnation and evaporation of the solvent, the samples were dried at 80 °C for 18 h. The nickel precursor was then thermally decomposed in air at 450 °C for 3 h using a heating rate of 1 °C/min. The resulting bimetallic materials were denoted **10Ni10Co/β** and **15Ni15Co/β**, respectively.

### 3.4. Characterization

The XRD patterns were recorded at room temperature using a Bruker D8 Advance diffractometer (Bruker AXS, Karlsruhe, Germany) at Bragg–Brentano geometry with CuKα radiation (λ = 1.5418 Å) and a LynxEye detector. The data was collected between 10 and 90 °2θ. Phase composition was determined with EVA v4 software with the reference ICDD-PDF2 database. The Topas-4.2 program was used to calculate the unit cell parameters, phase quantities, and mean crystallite sizes. The following reference cards were used: 00-047-0183 for zeolite β, 00-004-0850 for metallic Ni, 78-643 for NiO, 00-015-0806 for metallic Co and 00-042-1467 for Co_3_O_4_.

Nitrogen physisorption measurements with nitrogen were done at −196 °C with an AUTOSORB iQ-C-MP-AG-AG analyzer (Quantachrome Instruments, Anton Paar, Boynton Beach, FL, USA). The samples were outgassed at 350 °C under vacuum before performing the analysis. The amount of adsorbed nitrogen at 0.99 relative pressure was used for calculating the total pore volume by the Gurvich method.

Temperature-programmed reduction (TPR-TGA) was done using a STA449F5 Jupiter instrument (NETZSCH Gerätebau GmbH, Germany). Approximately 20 mg of catalyst was placed in the microbalance crucible and heated from ambient temperature to 600 °C at 5 °C/min under a flow of 5 vol.% H_2_ in Ar (100 cm^3^/min), followed by an isothermal period of 1 h. Before each TPR-TGA measurement, the sample was pretreated in situ in air at 500 °C for 1 h, using a heating rate of 10 °C/min.

XPS spectra were obtained using a SPECS Phoibos 100 X-ray photoelectron spectrometer operating in Fixed Analyzer Transmission (FAT) mode with a five-channel SPECS MCD-5 detector. An achromatic X-ray source SPECS XR50 was used with an Al/Mg X-ray tube. The X-ray was set to an Al Kα line at 12 kV and 200 W or a Mg Kα line at identical conditions. A flood gun SPECS FG22/35 was set up to minimize surface charge-related artifacts, and it was set to 1eV at 150 μA. Survey spectra were collected at pass energy 40 eV, and high-resolution spectra were collected at pass energy 15 eV and accumulated from 5 passes. The Mn 2p and Ni 2p spectra were also collected with prolonged dwell time to 1 s from the typical 0.2 s to reach a higher noise-to-signal ratio. The processing was made in CasaXPS (version 2.3.26, Casa Software Ltd., Devon, UK) with Shirley background and built in RSF. The binding energy in the spectra was set to 284.5 eV for C1s C-C bonds in adventitious carbon.

The acidity of the catalyst was investigated by FT-IR spectroscopy of adsorbed pyridine. Spectra were recorded by a Nicolet Compact 6700 type spectrometer (Thermo Scientific, Walthan, MA, USA). A special cell for in situ heat treatment and adsorption experiments was connected to the spectrophotometer. Thin, self-supported wafers were pressed from the catalyst of about 2 cm^2^ surface area. The samples were dehydrated at 350 °C in high vacuum before adsorbing 6 mbar of pyridine (Py) at 100 °C for 30 min. Pyridine was desorbed by evacuation at elevated temperatures, 100–200–300–400 °C for 30 min. Spectra were recorded with 2 cm^−1^ resolution with 128 scans. For quantitative comparison, the spectra were normalized to 5 mg/cm^2^ weight.

A qualitative and quantitative X-ray fluorescence (XRF) analysis was performed using a Spectro SpectroSCOUT (ED-XRF) spectrometer equipped with a rhodium X-ray tube, a silicon drift detector (SDD), and a sample chamber with vacuum and rotation capabilities. The installed vacuum chamber enables the qualitative and quantitative determination of chemical elements ranging from sodium (Na) to uranium (U). The samples to be analyzed must be in the form of a finely ground powder. The spectrometer uses automated software for semi-quantitative analysis, eliminating the need for standard measurements and calibration for most elements.

Hydrogen chemisorption experiments were performed using a Quantachrome Instrument AUTOSORB iQ-C-MP-AG-AG. In total, 200 mg of the sample was loaded into a U-shaped quartz reactor and placed between quartz wool plugs. The sample was pretreated first under nitrogen flow (50 mL/min) at 200 °C for 30 min with a heating rate of 10 degrees per minute, cooled down to 50 °C, and then reduced in situ under a flow of pure hydrogen (50 mL/min) at 500 °C for 1 h with a heating rate of 10 degrees per minute. After reduction, the sample was evacuated for 30 min at 500 °C. Afterwards, the material was cooled down to 40 °C, and evacuation took place for 30 min. The exposed active metal surface was calculated based on the hydrogen uptake. H/Ni and H/Co adsorption stoichiometric factors of 2 were used.

Solid-state NMR measurements were conducted on a Bruker Avance II+ 600 spectrometer (Bruker, Karlsruhe, Germany) operating at resonance frequencies of 600.01 MHz for ^1^H, 119.21 MHz for ^29^Si, and 156.34 MHz for ^27^Al. A 4 mm solid-state CP/MAS dual ^1^H/^31^P-^15^N probe was employed. Samples were loaded into 4 mm ZrO_2_ rotors and investigated under magic-angle spinning at 10 kHz for the ^29^Si measurements and 14 kHz for the ^27^Al measurements.

Quantitative ^29^Si NMR spectra were obtained using a single-pulse excitation sequence with a 90° pulse duration of 4.5 μs. The acquisition consisted of 3K time-domain points over a 29 kHz spectral width, with 244 scans and a 30 s relaxation delay between successive scans. The acquired data were zero-filled to 16K points and processed using an exponential window function prior to Fourier transformation.

The ^27^Al spectra were collected using single-pulse excitation with a 90° pulse duration of 2.8 μs. A total of 1024 scans were acquired using 128K time-domain points over a spectral width of 780 kHz, with a relaxation delay of 0.5 s.

For the ^1^H→^29^Si cross-polarization magic-angle-spinning (CP/MAS) experiments, a ^1^H excitation pulse of 3.6 μs, a contact time of 5 ms, and a relaxation delay of 5 s were employed. More than 4000 scans were accumulated, and ^1^H SPINAL-64 decoupling was applied during signal acquisition.

### 3.5. Catalytic Experiments

Before catalytic testing, the catalysts were reduced in an Ar/H_2_ atmosphere at 500 °C for 1 h. The temperature was increased at a rate of 5 °C/min. Catalytic conversion of levulinic acid (LA) was carried out in a high-pressure batch reactor. In total, 1 g of LA (Sigma-Aldrich, Darmstadt, Germany, 98%), 20 mL of water, and 0.2 g of catalyst were introduced into the reactor, corresponding to a catalyst-to-LA mass ratio of 0.2 g/g and an LA/H_2_O weight ratio of 1:20. The reactor was then pressurized with hydrogen to 30 bar and heated to 150 °C under continuous stirring at 700 rpm. The reaction was maintained at the target temperature for 4 h. The reaction products were analyzed by solution-state NMR spectroscopy, using a Bruker NEO 400 (work frequency for ^1^H—400.1 MHz) spectrophotometer, equipped with a 5 mm iProbe BBFO BBF/^1^H (Smart Probe) at 25 °C. To achieve a quantitative result, the samples were diluted with D_2_O and analyzed by single-pulse ^1^H experiment with 30° flip angle and 20 s. relaxation delay.

## 4. Conclusions

Mono- and bimetallic Co–Ni catalysts supported on nanosized β-zeolite were successfully synthesized via a post-synthesis impregnation method and evaluated for the catalytic hydrogenation of biomass-derived levulinic acid (LA) to γ-valerolactone (GVL), an important renewable platform chemical with potential applications as a sustainable fuel additive. The catalysts showed promising performance in the valorization of biomass-derived feedstocks, demonstrating the potential of zeolite-supported transition-metal catalysts for the production of value-added chemicals from renewable resources.

Among the investigated catalysts, the bimetallic Ni–Co/β-zeolite catalyst exhibited superior catalytic performance, achieving complete LA conversion (100%) and a high GVL yield of 92% at 150 °C, compared with the monometallic catalysts, and retained 98% conversion after three reaction cycles. The significantly enhanced catalytic activity can be attributed to the synergistic interaction between Ni and Co species, probably by the formation of the Ni-Co alloy phase. This may promote the dispersion of nickel, facilitating H_2_ activation and the sequential hydrogenation and cyclization steps involved in GVL formation.

The nanosized β-zeolite support also plays an important role in catalytic performance by providing a high surface area, a suitable pore structure, and accessible acid sites that can facilitate reactant adsorption and the transformation of reaction intermediates. The combination of metallic hydrogenation sites with the acidic functionality of the zeolite creates a potentially effective bifunctional catalytic system for the conversion of LA under relatively mild reaction conditions. The results indicate that catalytic performance is strongly influenced by both metal composition and metal–support interactions, highlighting the importance of rational catalyst design for efficient biomass conversion.

Overall, the high LA conversion and GVL yield achieved over the bimetallic Ni–Co/β-zeolite catalyst at a relatively moderate reaction temperature demonstrate its promising potential for LA valorization.

## Figures and Tables

**Table 1:** Physicochemical properties of the mono- and bimetallic zeolite β catalysts.

Samples	S_BET_ (m^2^/g)	S_micro_ (m^2^/g)	V_total_, (cm^3^/g)	V_micro_, (cm^3^/g)	Crystallite Size (nm)	Reduction Degree, %
Zeolite β	758	580	0.83	0.23	-	-
10Co/β	606	475	0.69	0.19	n.d.	34.0
10Ni/β	636	514	0.73	0.20	n.d.	100.0
15Co/β	550	421	0.59	0.17	26	85.4
15Ni/β	511	401	0.52	0.16	35	91.5
10Ni10Co/β	510	405	0.64	0.16	11-Co_3_O_4_; 11-NiO	75.7
15Ni15Co/β	414	323	0.42	0.13	21-Co_3_O_4_; 14-NiO	65.7

**Table 2:** Areas of the signals of the different Si structural units, obtained by deconvolution of the spectral patterns in the single-pulse ^29^Si NMR spectra.

Sample	Si(2OH)		Si(2Al) + Si(1OH)		Si(1Al)		Si(0Al)					
	ppm	%	ppm	%	ppm	%	ppm	%	ppm	%	ppm	%
β parent	−98	3	−102	13	−105	22	−110	49	−114	13		
10Ni/β	−97	3	−101	10	−105	18	−110	54	−114	9	−116	5
	−92	1										
15Ni/β	−92	2	−100	8	−105	23	−111	46	−115	14	−120	7
10Co/β	−94	1	−99	8	−103	17	−110	49	−114	17	−122	8
15Co/β	−92	1	−96	3	−103	16	−110	63	−115	13	−120	4
10Ni10Co/β	−98	9	−102	20	−106	13	−110	40	−115	9	−119	7
	−91	2										
15Ni15Co/β	−98	3	−102	11	−106	17	−111	37	−115	16	−119	15
	−93	1										

**Table 3:** Chemical composition of 10Ni10Co/β and 15Ni15Co/β based on the XPS data.

Catalysts	O	Si	Al	Ni	Co
	at.%				
10Ni10Co/β	66.8	27.4	1.2	3.7	0.9
15Ni15Co/β	62.2	21.0	1.9	13.4	1.5

**Table 4:** Conversion of levulinic acid and product yields (Y) at 150 °C on the mono- and bimetallic Ni/Co β-supported catalysts.

Samples	LA Conversion mol.%	GVL Yield mol.%	4-Hydroxy-pentanoic Acid Yield mol.%
10Co/β	2.0 (1.0 *)	2.0	-
10Ni/β	12.6 (5.6 *)	11.5	1.1
15Co/β	10.0 (3.8 *)	9.0	1.0
15Ni/β	24.8 (10.0 *)	23.0	1.8
10Ni10Co/β	53 (50.0 *)	48.0	5.0
15Ni15Co/β	100 (98.0 *)	92.0	8.0

* Catalytic conversion in three reaction cycles.

## Data Availability

The original contributions presented in this study are included in the article and Supplementary Materials. Further inquiries can be directed to the corresponding author.

## Supplementary Materials

The following supporting information can be downloaded at: https://www.mdpi.com/article/10.3390/molecules31214015/s1, Figure S1: ^29^Si NMR spectrum of parent zeolite β with the results of the deconvolution; Figure S2: ^1^H-^29^Si CPMAS (red) and direct excitation ^29^Si spectra (black) of parent zeolite β; Figure S3: ^27^Al NMR spectrum of parent zeolite β; Figure S4: Pressure dependence of hydrogen uptake on the studied samples; Table S1: Brønsted and Lewis acidity of catalysts studied, based on FT-IR bands of adsorbed pyridine; Table S2: Chemisorption data and catalytic activity calculated as TOF for the studied samples; Table S3: Chemical composition of the fresh and spent catalysts from XRF measurements; Table S4. Catalytic performance of comparable catalysts reported in the literature.
