# Supplementary material for: Hydrodeoxygenation of Levulinic Acid to γ-Valerolactone over Nano-β Zeolite-Supported Co–Ni Catalysts
Source: Molecules. 2026 Nov 6;31(21):4015. doi: 10.3390/molecules31214015 (PMC13764914; doi:10.3390/molecules31214015)
Supplement: Supplementary file 1 [file molecules-31-04015-s001.zip › molecules-4606658-supplementary.pdf]

Supplementary Materials

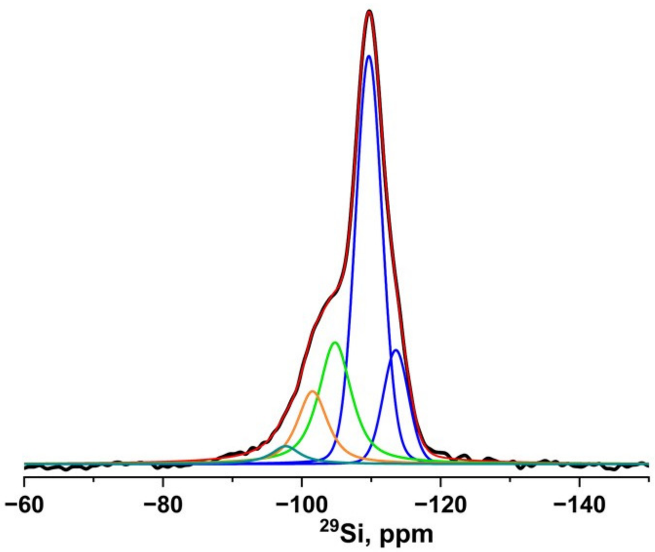

Figure S1. ^{29}Si NMR spectrum of parent zeolite β with the results of the deconvolution. Black line represents the experimental spectrum, red line – the overall fit, the individual components are presented with different colors: (Si(0Al) blue; Si(1Al) – green; Si(2Al) and Si(1OH) – light brown, minor components – dark green

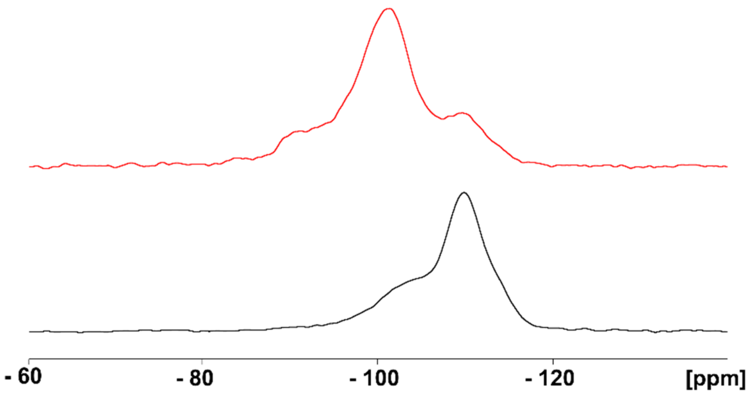

Figure S2. ^{1}H-^{29}Si CPMAS (red) and direct excitation ^{29}Si spectra (black) of parent zeolite-β.

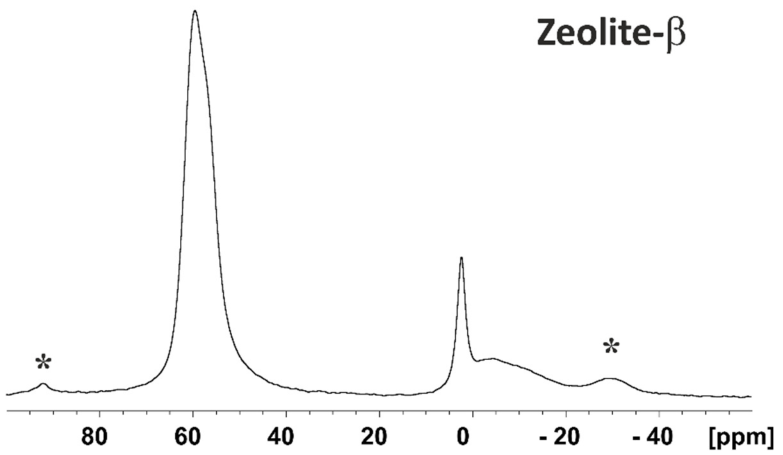

Figure S3. ^{27}Al NMR spectrum of parent zeolite β, spinning side bands are denoted with asterisk.

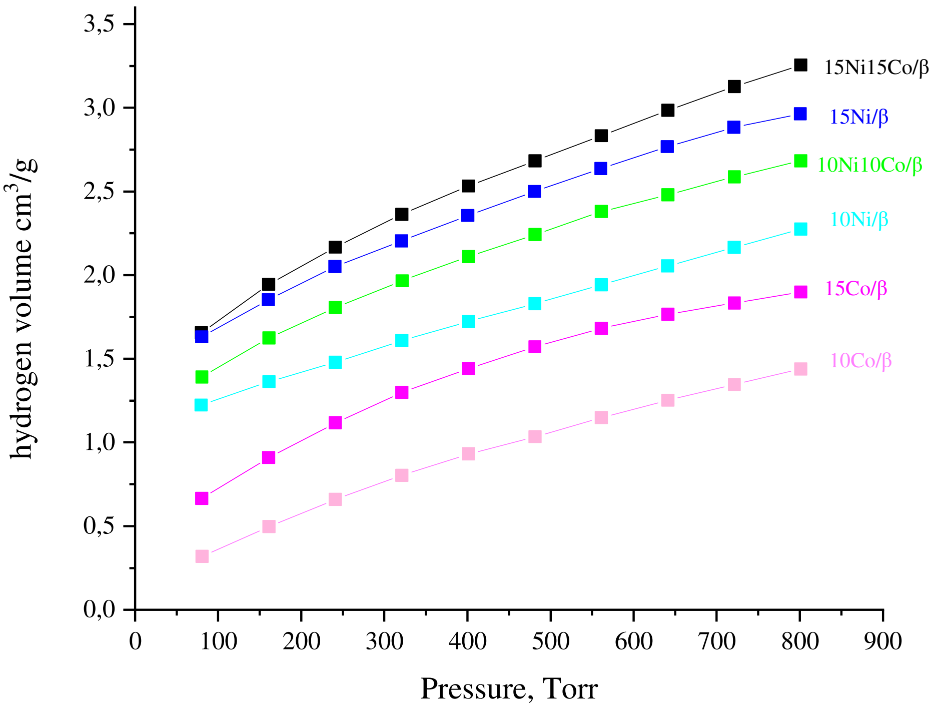

Figure S4. Pressure dependence of hydrogen uptake on the studied samples

Table S1. Brønsted and Lewis acidity of catalysts studied, based on FT-IR bands of adsorbed pyridine

Samples	Py des. temp., °C	n_{Py} (B)* mmol/g[a]	n_{Py} (L)* mmol/g[b]	L/B
β	200	0.396	0.283	0.71
	400	0.149	0.340	2.28
15Ni/β	200	0.205	0.378	1.85
	400	0.053	0.395	7.69
10Ni10Co/β	200	0.188	0.500	2.70
	400	0.012	0.436	33.30
15Ni15Co/β	200	0.120	0.411	3.45
	400	0.021	0.334	15.90

[a]calculated by the integrated area of 1547 cm^{-1} band; [b]calculated by the integrated area of 1451/1455 cm^{-1} band; *n_{Py}=A/εS, where A is the integrated area of corresponding FT-IR band (cm^{-1}), ε is the molar absorption coefficient (cm/μmol), 1.07 for B and 1.65 for L bands [41], and S is the thickness of normalized pellet: 5 mg/cm^2.

Table S2. Chemisorption data and catalytic activity calculated as TOF for the studied samples.

Catalysts	Dispersion, %	Hydrogen uptake, μmol/g	Metal surface area*, m^2/g	Metal surface area**, m^2/g	Ni particles, nm	LA conversion, mol. %	TOF, s^{-1}
10Ni/β	5.94	50.58	3.96	39.57	17.0	12.6	0.00745
15Ni/β	5.48	70.02	5.48	36.52	18.4	24.8	0.01059
10Co/β	1.63	13.80	1.10	11.01	61.2	3.0	0.00650
15Co/β	2.52	32.04	2.56	17.04	39.6	10.0	0.00933
10Ni10Co/β	7.06	60.12	4.70	47.03	14.3	53.0	0.02636
15Ni15Co/β	5.68	72.57	5.68	37.85	17.8	100.0	0.04120

* calculated per gram sample; ** calculated per gram metal, TOF= r_{LA}/n_{Ni}, s^{-1}, where r_{LA} is LA consumption rate, n_{Ni} is the number of accessible Ni surface sites determined by H_2 chemisorption.

Table S3. Chemical composition of the fresh and spent catalysts from XRF measurements

Catalysts	Al, %	Si, %	Ni, %	Co, %
15Ni/β	1.80	31.67	16.53	-
Spent 15Ni/β	1.77	30.97	17.26	-
15Co/β	1.63	31.90	-	16.47
Spent 15Co/β	2.44	39.80	-	7.76
10Ni10Co/β	1.60	30.32	8.72	9.36
Spent 10Ni10Co/β	2.10	37.01	7.14	3.76
15Ni15Co/β	1.30	22.65	11.87	14.18
Spent 15Ni15Co/β	1.78	26.75	10.49	10.98

Table S4. Catalytic performance of comparable catalysts reported in the literature.

Catalysts	Reaction conditions	Catalytic performance	References
Ru catalyst + acidic co-catalyst (Amberlyst A70)	70 °C, 0.5 MPa H_2, 3 h	99 mol% GVL yield; activity of 558 h^{-1}	[27]
Co/Al_2O_3	180 °C, 5 MPa H_2, 3 h; 30 mL solvent	Complete LA conversion; selectivity to GVL – >99%	[28]
Ni-ZSM-5	Catalyst ~ 0.1 g, GHSV = 8.166 mL s^{-1} $gcat^{-1}$, 300 ◦C.	Conversion – 70.4%, selectivity to GVL - 61.8 %	[43]
5% Pt/carbon	H_2 pressure-1 bar, 265 °C, 10 wt.% LA in 1, 4-dioxane and WHSV of 0.512 h^{-1}, 50 h	Conversion – 100 %, selectivity to GVL – 98.6 %	[44]
Ni_1Cu_1/SiO_2	Catalyst-0.2 g, 275 ◦C	Conversion – 80 %, selectivity to GVL – 90.3 %	[45]
Ni_5Mo_1-MOF	LA (1 mmol), catalyst 0.1 g, water (10 mL), 160 ◦C, 2.5 h, 2 MPa H_2	Conversion – 100 %, selectivity to GVL – 100 %	[46]
Ni-Co/γ-Al_2O_3	Catalyst 1 g, WHSV: 1.14 h^{-1}, 250 ◦C, 1 atm, H_2/LA: 8 (molar ratio)	Conversion – 100 %, selectivity to GVL – 100 %	[47]
15Ni15Co/β	1 g LA, 20 mL water, 0.2 g catalyst; catalyst/LA = 0.2 g/g; LA/H_2O (wt.) = 1:20, 150◦C, 30 atm	Conversion – 100 %, selectivity to GVL – 92 %	This work
